# Depression-like effects induced by chronic unpredictable mild stress in mice are rapidly reversed by a partial negative allosteric modulator of mGlu_5_ receptor, M-5MPEP

**DOI:** 10.1007/s00213-024-06724-4

**Published:** 2024-11-30

**Authors:** Agnieszka Pałucha-Poniewiera, Bartosz Bobula, Anna Rafało-Ulińska, Katarzyna Kaczorowska

**Affiliations:** 1 Department of Neurobiology, Maj Institute of Pharmacology, Polish Academy of Sciences, 12 Smętna Street, Kraków, 31-343 Poland; 2 Department of Physiology, Maj Institute of Pharmacology, Polish Academy of Sciences, 12 Smętna Street, Kraków, 31-343 Poland; 3 Department of Medicinal Chemistry, Maj Institute of Pharmacology, Polish Academy of Sciences, 12 Smętna Street, Kraków, 31-343 Poland

**Keywords:** Antidepressant, CUMS, eEF2, LTP, M-5MPEP, mGlu_5_ receptor, mTOR, RAAD, Sucrose preference, TrkB

## Abstract

**Rationale:**

Due to the numerous limitations of ketamine as a rapid-acting antidepressant drug (RAAD), research is still being conducted to find an effective and safe alternative to this drug. Recent studies indicate that the partial mGlu_5_ receptor negative allosteric modulator (NAM), 2-(2-(3-methoxyphenyl)ethynyl)-5-methylpyridine (M-5MPEP), has therapeutic potential as an antidepressant.

**Objectives:**

The study aimed to investigate the potential rapid antidepressant-like effect of M-5MPEP in a mouse model of depression and to determine the mechanism of this action.

**Methods:**

Chronic unpredictable mild stress (CUMS) was used as an animal model of depression. The effects of single and four-day administration of M-5MPEP on CUMS-induced animal behaviors reflecting anhedonia, apathy, and helplessness were studied. Western blot was applied to measure the levels of proteins potentially involved in a rapid antidepressant effect, including mammalian target of rapamycin (mTOR), eukaryotic elongation factor 2 (eEF2), tropomyosin receptor kinase B (TrkB), and serotonin transporter (SERT), both in the hippocampus and the prefrontal cortex (PFC). Furthermore, excitatory synaptic transmission and long-term potentiation (LTP) were measured in the medial PFC (mPFC).

**Results:**

We showed that M-5MPEP administration for four consecutive days abolished CUMS-induced apathy- and anhedonia-like symptoms in a mouse model of depression. We also found that these effects were accompanied by changes in hippocampal TrkB levels and mTOR and eEF2 levels in the PFC. Using electrophysiological techniques, we showed that the four-day M-5MPEP treatment reversed chronic stress-induced increases in excitatory synaptic potential and CUMS-impaired LTP in the mPFC.

**Conclusions:**

Partial mGlu_5_ receptor NAM, M-5MPEP, appears to be a potentially effective new RAAD and deserves further study.

**Supplementary Information:**

The online version contains supplementary material available at 10.1007/s00213-024-06724-4.

## Introduction

Due to limited effectiveness and the numerous disadvantages of using antidepressant drugs (ADs) working as monoaminergic modulators, new ways of treating depression are still being sought. Targeting the glutamatergic system has been brought forward as a promising strategy to develop genuinely novel ADs (Skolnick et al. 2009). Numerous studies showing the rapid antidepressant effect of subanesthetic doses of the NMDA receptor antagonist ketamine have strongly supported this theory (Berman et al. 2000; Lapidus et al. 2014; Zarate et al. 2006). However, the adverse effects of ketamine (aan het Rot et al. 2010; Krystal et al. 1994) still indicate the need to search for an AD based on the modulation of glutamatergic transmission, which could be safer than ketamine and, at the same time, would share its effectiveness and speed of action (Pałucha-Poniewiera 2018).

Preclinical studies have shown that one of the metabotropic glutamate (mGlu) receptors, namely the mGlu_5_ receptor, could become the target of a new antidepressant therapy. It has been found that antagonists and negative allosteric modulators (NAMs) of this receptor (e.g., MTEP, MPEP) have therapeutic potential in animal models of depression and screening tests (Chaki et al. 2013; Fuxe and Borroto-Escuela 2015; Kato et al. 2015; Pałucha et al. 2005). Involvement of the mGlu_5_ receptor in the pathophysiology of depression has also been suggested (Deschwanden et al. 2011). Unfortunately, several undesirable effects, including psychotomimetic actions and cognitive dysfunctions, inhibited further attempts to introduce these compounds into the clinic (Homayoun et al. 2004; Kinney et al. 2003). Recent studies indicate that partial mGlu_5_ receptor NAMs, which do not entirely block the effect of mGlu_5_ receptor activation despite the complete occupation of its allosteric site, maybe a promising alternative in this indication. One of these compounds, namely 2-(2-(3-methoxyphenyl)ethynyl)-5-methylpyridine (M-5MPEP), has been shown to demonstrate high therapeutic effectiveness in animal studies, including potential antidepressant-like effects (Gould et al. 2016; Holter et al. 2021) and at the same time is devoid of adverse effects typical of full mGlu_5_ NAMs, including psychotomimetic effects (Gould et al. 2016).

New important information on the potential antidepressant action of M-5MPEP has been provided in our recent study, which showed that M-5MPEP-induced antidepressant-like effect in mice was fast and sustained, similar to the effect of rapid-acting antidepressant drugs (RAADs) like ketamine (Pałucha-Poniewiera et al. 2024). In particular, we demonstrated that M-5MPEP caused a dose-dependent antidepressant-like action in the tail suspension test (TST) 60 min after injection, and this effect was antagonized by an AMPA receptor antagonist (NBQX) and a tropomyosin receptor kinase B (TrkB) receptor antagonist (ANA-12) but not by 5HT_1A_ and 5HT_2A/2C_ receptor antagonists. Furthermore, short-term, four-day administration of M-5MPEP (30 mg/kg) induced prolonged antidepressant-like actions characteristic of RAAD (24 h after administration) in the splash test and the TST, which were also reversed by ANA-12, suggesting the involvement of the TrkB/brain derived neurotrophic factor (BDNF) signaling in the sustained M-5MPEP effects (Pałucha-Poniewiera et al. 2024).

Although these data suggested the M-5MPEP-induced rapid, ketamine-like antidepressant effect, it must be remembered that this study was performed using a screening test. It did not include effects in a depression model that would allow the dynamics of therapeutic effects to be observed and tracked. Therefore, we decided to investigate the effect of M-5MPEP on the behavioral and cellular effects of chronic unpredictable mild stress (CUMS) in mice, a well-validated and established mouse model of depression (Willner 2017). We examined the effects of a single and four-day administration of M-5MPEP (30 mg/kg) on CUMS-induced behavioral effects, such as anhedonia, as measured by a preference for consuming 1% sucrose (Willner et al. 1992), apathy-like state, measured as grooming time in the splash test (Cathomas et al. 2015; Pałucha-Poniewiera et al. 2020), and behavioral despair in the TST.

The next goal was to investigate the mechanisms related to the behavioral effects obtained in the CUMS model. For this purpose, we relied on two main theories explaining the mechanism of the fast antidepressant effect based on studies on ketamine. Both hypotheses assume a crucial role of the rapid regulation of protein synthesis, enabling the intensification of neuroplasticity processes in the brain areas associated with emotions and the involvement of the neurotrophic factor BDNF (Kim et al. 2023).

The first hypothesis, called the disinhibition theory assumes that ketamine’s blockade of NMDA receptors on interneurons causes disinhibition of pyramidal neurons in the PFC, enhanced glutamatergic transmission, and subsequent activation of the mTOR pathway responsible for the synthesis of proteins associated with the intensification of neuroplasticity processes (e.g., GluA1, PSD-95, Synapsin-1, BDNF) (Li et al. 2010). The second theory says that blocking NMDA receptors by ketamine in action-potential-independent conditions in the hippocampus leads to the inhibition of eEF2 phosphorylation, which results in rapid local protein translation, including BDNF protein synthesis, which in turn induces a specific form of synaptic potentiation, namely homeostatic synaptic plasticity (Autry et al. 2011; Kim et al. 2023). Importantly, these processes, initiated in the hippocampus, have been proposed to be executed in the PFC, which is considered involved in the sustained and cumulative effects of ketamine (Kim et al. 2023). Based on the above hypotheses, we decided to investigate possible changes in mTOR and eEF2 proteins and TrkB receptor protein (a receptor for BDNF) in the hippocampus and PFC in CUMS animals after M-5MPEP administration. Furthermore, the serotonergic transporter (SERT) protein level was examined in both the hippocampus and PFC based on the reported involvement of the 5-HT system in the mechanism of action of RAADs (du Jardin et al. 2016; Gigliucci et al. 2013).

Since depressive symptoms are associated with changes in neuroplasticity in glutamatergic cortico-limbic brain regions, including the PFC (Pittenger and Duman 2008; Thompson 2023), and the antidepressant effect of RAADs has been proposed to correlate with the modulation of glutamatergic transmission and the enhancement of neuroplasticity processes in this structure (Li et al. 2010; Mingardi et al. 2023; Sala et al. 2022), we also decided to examine the effect of M-5MPEP treatment on CUMS-induced changes in glutamatergic transmission and the effect on stress-induced impairments of neuroplasticity-dependent process of long-term potentiation (LTP) in the mPFC, using electrophysiological methods. Based on our recent study showing that the CUMS-induced LTP disturbance in the PFC in mice was reversed by *(R)*-ketamine combined with a mGlu_2/3_ receptor antagonist, used as an effective RAAD (Pałucha-Poniewiera et al. 2023), we hypothesize that the improvement of the chronic stress-induced LTP impairment in this brain structure may be responsible for the antidepressant-like effect of M-5MPEP in this depression model.

## Methods

### Animals and housing

The experiments were performed on male C57BL/6J mice (Charles River, Germany). Animals were maintained under standard laboratory conditions in terms of temperature (22 ± 2 °C), humidity (55 ± 10%), and lighting (light phase 7:00–19:00) with free access to food and tap water. The mice were seven weeks of age at the beginning of the experiment and were divided into two groups: control mice (non-stressed, designated NS) and mice subjected to CUMS. Each experimental group consisted of ten to twelve animals. The tests were performed by a person unaware of the individual’s affiliation to the experimental group. All procedures were conducted following the National Institutes of Health Animal Care and Use Committee guidelines and were approved by the Second Local Ethics Committee in Kraków, Poland (permission number: 154/2023). The three Rs principles were applied in the planning and execution of the experiments. Every effort was made to reduce the number of animals used and to avoid and minimize animal suffering.

### Compounds and treatment

M-5MPEP (2-[2-(3-methoxyphenyl)ethynyl]-5-methylpyridine) was synthesized in the Department of Medicinal Chemistry, Maj Institute of Pharmacology Polish Academy of Sciences, by K.K. (compound synthesis and structure confirmation can be found in the Supplementary file). M-5MPEP and *(S)*-(+)-ketamine hydrochloride (Tocris Cookson, Ltd., Bristol, UK), UK) were diluted in a suspension of 0.5% methylcellulose/0.9% NaCl, which was used as a vehicle. All compounds and vehicles were injected intraperitoneally (i.p.) at a constant 10 ml/kg volume. Doses and times of drug administration were determined based on our own previous research and literature data.

### CUMS and behavioral tests

**Fig. 1:**
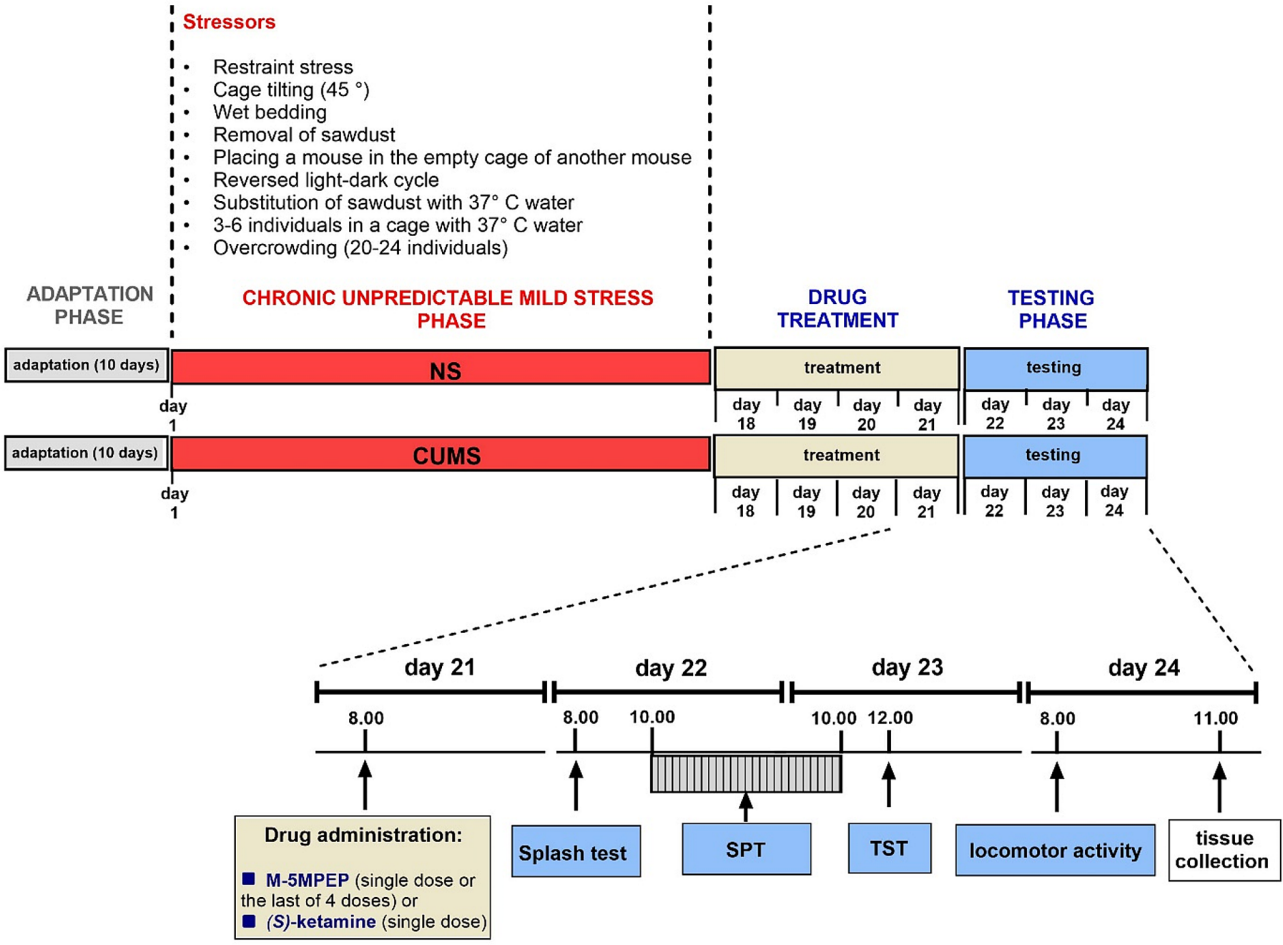
The schedule of the CUMS experiments. After ten days of adaptation, the animals were subjected to the CUMS procedure. Two stressors from those given in the scheme were applied daily, with a two-hour break between stressors. The NS group was not subjected to any procedures during this time. On the 18th day from the beginning of CUMS, drug administration started in the 4-day application group. On the 21st day, the animals were given the last of four doses of M-5MPEP or a single dose. In a reference group, a single dose of *(S)*-ketamine was given on the 21st day from the beginning of CUMS. After twenty-four hours, the splash test was performed, and the SPT started 2 h later. The next day, the TST was applied, and on the subsequent day, the locomotor activity was measured, after which the animals were handed over for tissue collection

The CUMS procedure was performed based on our experience and previously published experimental schedules with the necessary modifications (Pałucha-Poniewiera et al. 2020, 2021). The detailed experimental schedule, including the adaptation stage, chronic unpredictable mild stress, administration of test compounds, and subsequent behavioral experiments, is presented in Fig. 1.

The following procedures were used in the behavioral testing phase:

### Splash test

The splash test was performed as described previously (Pałucha-Poniewiera et al. 2021) with minor modifications. Briefly, animals were adapted for the experimental room for 30 min. The test was performed under dimmed lighting. A high-viscosity 10% sucrose solution (approximately 0.2 ml) was sprayed on the dorsal coat of the mice to stimulate self-grooming behavior. Then, the duration of grooming was manually recorded for five minutes by an experimenter blind to the treatment.

### Sucrose preference test (SPT)

The SPT was performed as previously described (Pałucha-Poniewiera et al. 2021). For 24 h, the animals could choose between one of the two identical bottles. The first bottle contained a 1% sucrose solution, and the second contained tap water. The position of the bottles was switched 12 h after the start of the experiment. At the beginning and end of the test, the bottles were weighed, and the liquid consumption was calculated. The preference for sucrose consumption was calculated as a ratio of the consumed sucrose solution to the total amount of liquid consumed.

### Tail suspension test

The experiments were carried out as previously described (Pałucha-Poniewiera et al. 2020). The mice were habituated to the testing room for 30 min before the experiments. Each mouse was attached by its tail to the table’s edge with adhesive tape. The total duration of immobility was manually measured for six minutes by an experimenter blind to the treatment.

### Locomotor activity

12-station photobeam activity system (Opto Varimex 4, Auto Track System 4.41, Columbus Instruments, Columbus, OH, USA) equipped with Plexiglas locomotor activity chambers (40 × 40 × 40 cm) was used to measure the locomotor activity of the mice. After placing the animals individually in the locomotor activity chambers, the total distance traveled during a 30-minute experimental session was measured and stored every 3 min.

### Tissue preparation

After completing the behavioral tests, the animals were sacrificed by dislocating the cervical vertebrae. Then, the mice were decapitated, and the tissue was collected for further research (Western blot and Elisa). The animals intended for electrophysiological studies were anesthetized using isoflurane (Aerrane, Baxter, Deerfield, IL, USA).

### Synaptosome preparation and Western blotting

Tissue samples were dissected from the PFC and hippocampus and frozen on dry ice. After thawing on ice, the samples were homogenized in ice-cold lysis buffer [0.32 M sucrose, 20 mM HEPES (pH 7.4), 1 mM EDTA; 1 × protease inhibitor cocktail, 5 mM NaF, and 1 mM NaVO3]. Homogenates were centrifuged at 2800 rpm for 10 min at 4 °C, and the resulting supernatant was centrifuged at 12,000 rpm for 10 min at 4 °C. The obtained pellets were then sonicated in protein lysis buffer (50 mM Tris-HCl (pH 7.5), 150 mM NaCl, 1% Triton X-100, 0.1% SDS, 2 mM EDTA, 1 mM NaVO3, 5 mM NaF, and protease inhibitor cocktail). BCA kit (Thermo Scientific, USA) was used to measure protein concentrations. The proteins were separated by SDS-PAGE and transferred to nitrocellulose membranes. 1% of the blocking solution (BM Chemiluminescence Western Blotting Kit (Mouse/Rabbit) made by Roche, Switzerland) was used to block for 1 h. Then, the membranes were incubated overnight at 4 °C with the primary antibodies: Anti-mTOR (mTOR 1:1000; Cell Signaling Technology, USA), Anti-phospho-mTOR (pmTOR, S2481, 1:1000; Abcam, USA), Anti-eEF2 (eEF2 1:1000; Abcam, USA), Anti- phospho-eEF2 (pheEF2 (phospho T56) 1:1000; Abcam, USA), Anti-SERT (SERT 1:1000; Sigma-Aldrich, Germany), Anti-TrkB (TrkB 1:1000; Cell signaling Technology, USA). Afterward, the membranes were washed three times for 10 min using Tris-buffered saline with Tween (TBS-T) and incubated for 60 min with secondary antibodies (anti-mouse or anti-rabbit-IgG-peroxidase-conjugated antibodies Vector Laboratories, USA). After incubation, the membranes were washed thrice for 10 min with TBS-T and incubated with a detection reagent (Bio-Rad, USA). Fuji-Las 1000 system, equipped with Fuji Image Gauge v.4.0 software, was used to visualize and measure the signal. A primary monoclonal antibody, Glyceraldehyde 3-phosphate dehydrogenase (GAPDH, 1:500; Millipore, Germany), was used to check the transfer and loading. The final result is the ratio of particular proteins’ optical density to GAPDH’s optical density.

### Measurement of BDNF concentration

ELISA kit purchased from R&D Systems, Inc. Minneapolis, MN, USA (Catalog Number SBNT00) was used to measure the total BDNF concentrations. The samples were thawed on ice and 100-fold diluted in Calibrator Diluent RD5K. A 96-well microtiter plate containing 50 µl of Assay Diluent RD1-123 and 50 µl of each sample or standard was incubated for two hours at room temperature with vigorous mixing (550 rpm, ThermoMixer C, Eppendorf, Hamburg, Germany). After rinsing each well four times with 400 µl of wash buffer, an enzyme-linked monoclonal antibody specific for BDNF was added (200 µl) to the wells and incubated at room temperature for one hour. Then, the wells were washed four times with 400 µl of wash buffer. Then, 200 µl of substrate solution was added and incubated in the dark for one hour. The plates were read at 450 and 540 nm using a spectrophotometer (Synergy HTX multimode reader machine; BioTek Instruments Inc., Winooski, VT, USA). The data were linearized by plotting the total BDNF concentrations log versus the O.D log. The total BDNF concentration was normalized to the protein concentration of each sample.

### Electrophysiological experiment

#### Slice preparation

Under anesthesia conditions, the brains were rapidly removed from the skulls and prepared in NMDG-based, cold artificial cerebrospinal fluid according to the procedure described by Ting et al. (2018). Cortical slices (380 μm thick) obtained from the medial prefrontal cortex (mPFC) were cut in the coronal plane using a Leica VT 1000s vibrating microtome. The slices were incubated in carbogen bubbled ACSF containing (in mM) NaCl (132), NaHCO_3_ (26), CaCl_2_ (2.5), D-glucose (10), KCl (5), MgSO4 (1.3), and KH_2_PO_4_ (1.25) at 32 ± 0.5 °C, then transferred to the recording chamber (interface type) and superfused (2.5 mL/min) with ACSF containing (in mM) NaCl (132), NaHCO_3_ (26), CaCl_2_ (2.5), D-glucose (10), KCl (2), MgSO_4_ (1.3), and KH_2_PO_4_ (1.25).

### Field potential recording and long-term potentiation (LTP) induction

A stimulating electrode (concentric, Pt-Ir; FHC, USA) was placed in cortical layer V. Basic stimulation (0.016 Hz frequency, duration of 0.2 ms) was applied using a constant-current stimulus isolation unit (WPI). Field potential (FP) recordings were performed using ACSF-filled glass micropipettes (1–2 MΩ). Glass electrodes were placed in cortical layer II/III. The recorded responses were amplified (EXT 10–2 F amplifier, NPI), then filtered (1 Hz-1 kHz), A/D converted (10 kHz sampling rate), and collected on a commercial personal computer with Micro1401 interface and Signal 4 software (CED). An input-output (stimulus-response) curve was made for every single slice. Stimulation intensity was increased stepwise from 0 to 100 µA with 5 µA steps to obtain the curve. One response was recorded at each stimulation intensity. Then, the stimulation intensity was adjusted to evoke a response of 30% of the maximum amplitude.

For LTP induction, theta burst stimulation (TBS) was used. TBS was composed of 10 stimuli trains at 5 Hz, repeated 5 times with 15-s gaps. The single train consisted of five 100 Hz pulses. The single pulse duration was increased from 0.2 to 0.3 ms.

For each slice, the stimulus-response data were fit with the Boltzmann equation: Vi = Vmax/(1 + exp((u − uh)/−S), where Vmax is the maximum FP amplitude; u is the stimulation intensity; uh is the stimulation intensity evoking FP of half-maximum amplitude; and S is the factor proportional to the slope of the curve.

### Data analysis

All the results obtained were expressed as the mean ± standard error of the mean (SEM). GraphPad Prism 7.00 (GraphPad Software, San Diego, CA, USA) was used to analyze behavioral data. The effects obtained in the splash test, SPT, and TST were analyzed using one-way ANOVA followed by Tukey’s post hoc test. Locomotor activity data were evaluated by repeated-measures ANOVA. The results obtained using Western blot and the ELISA method were analyzed by one-way ANOVA followed by Tukey’s multiple comparisons tests using GraphPad Prism version 9.2.0 for Windows (GraphPad Software, San Diego, CA, USA). Statistical analysis of the electrophysiological data was done using two-way ANOVA followed by Tukey’s post hoc test using GraphPad Prism 4.00 (GraphPad Software, San Diego, CA, USA). The results were considered to be significant if the *p*-values were below 0.05.

## Results

### Behavioral effects of a single and four-day administration of M-5MPEP in the CUMS model of depression

**Fig. 2:**
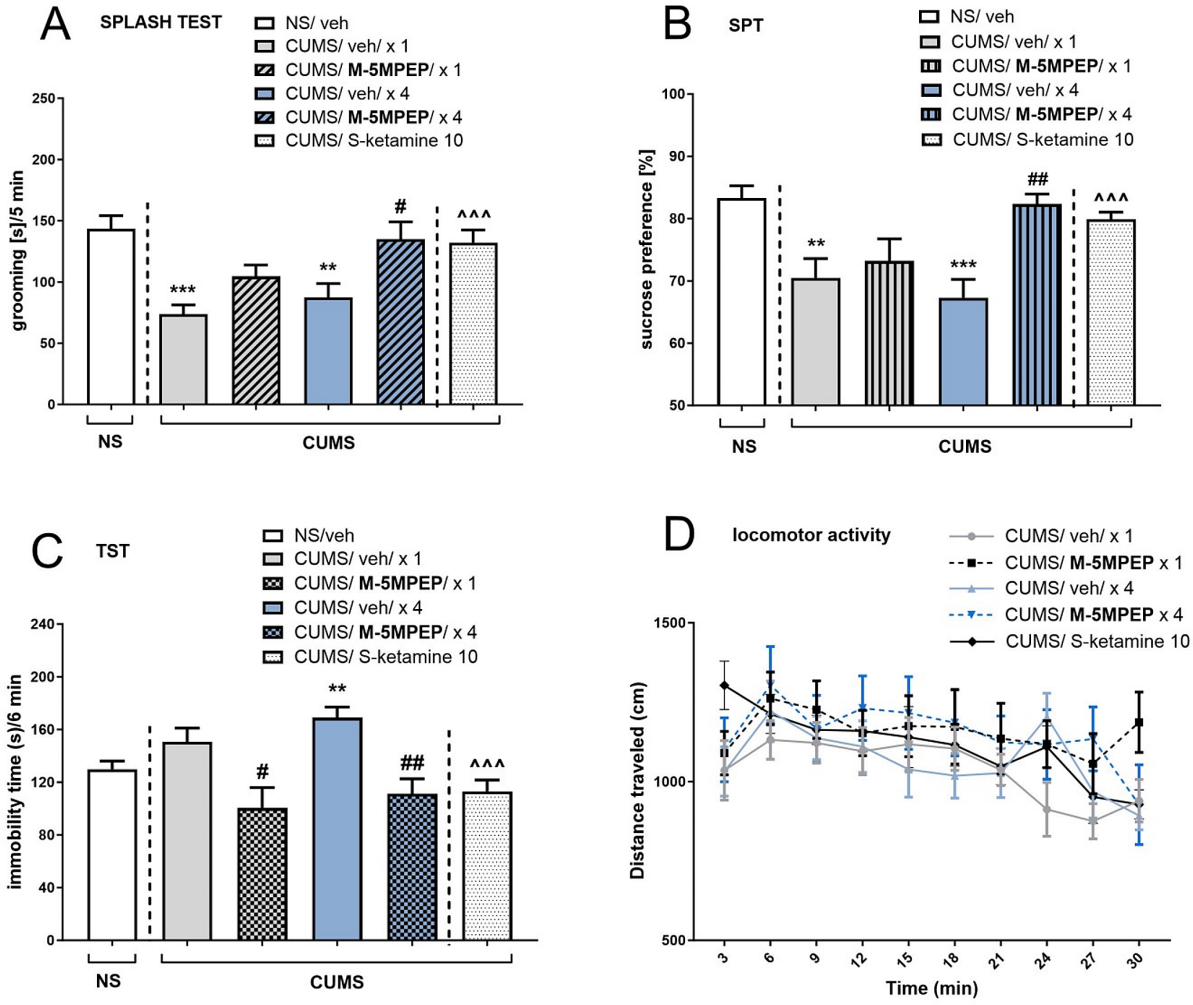
The antidepressant-like effects of single and four-day administration of M-5MPEP (30 mg/kg) in the CUMS model of depression. (**A**) M-5MPEP effect in the splash test; (**B**) M-5MPEP effect in the sucrose preference test; (**C**) M-5MPEP effect in the TST; (**D**) M-5MPEP effect in the locomotor activity test. The values are expressed as the means ± SEM and were analyzed by one-way ANOVA followed by Tukey’s post hoc test (**A**, **B**, **C**) or repeated-measures ANOVA (**D**). ** *p* < 0.01, *** *p* < 0.001 vs. the NS group; # *p* < 0.05, ## *p* < 0.01 vs. a respective CUMS/vehicle group; ^^^ *p* < 0.001 vs. CUMS/vehicle/x 1 group (*t*-test) (*n* = 10–12)

In the splash test, CUMS induced a significant reduction in grooming time compared to NS vehicle-treated groups, both for single and four-day vehicle injections (*t*-test, *p* < 0.001 and *p* < 0.01, respectively; Fig. 2A). In the stressed mice, one-way ANOVA showed an effect of M-5MPEP (30 mg/kg) [F(3,36) = 5.84, *p* < 0.01]. Tukey’s post hoc test revealed that a four-day M-5MPEP treatment was effective in reversing CUMS-induced apathy-like effects (*p* < 0.05), while a single M-5MPEP administration was ineffective in this test (*p* > 0.05). *(S)*-ketamine (10 mg/kg), used as a reference drug, increased grooming time in the splash test compared to vehicle-treated CUMS mice (*t*-test, *p* < 0.001; Fig. 2A).

CUMS produced a reduction in a sucrose preference in the SPT, both in a single and four-day vehicle-treated group (*t*-test, *p* < 0.01 and *p* < 0.001, respectively; Fig. 2B). One-way ANOVA revealed the main effect of M-5MPEP (30 mg/kg) in the stressed mice [F(3,35) = 4.601, *p* < 0.01] and Tukey’s post hoc test showed that four-day M-5MPEP treatment was required to reverse CUMS effects in the SPT (*p* < 0.01). *(S)*-ketamine (10 mg/kg) used as a reference drug also reversed CUMS-induced reduction in the sucrose preference (*t*-test, *p* < 0.001; Fig. 2B).

An influence of CUMS on the behavior of the mice was also observed in the TST, where vehicle-treated CUMS mice (four-day vehicle treatment) revealed an increase in the immobility time compared to NS controls (*t*-test, *p* < 0.01; Fig. 2C). In the group of single vehicle treatment, a tendency to increase this parameter was observed (*t*-test, *p* > 0.05, Fig. 2C). One-way ANOVA showed the M-5MPEP (30 mg/kg) effect in the CUMS mice [F(3,33) = 7.195, *p* < 0.001]. Tukey’s post hoc test revealed that both single and four-day M-5MPEP treatment reduced the immobility time of mice compared to a respective vehicle-treated control group (*p* < 0.05 and *p* < 0.01, respectively). A reference drug, *(S)*-ketamine (10 mg/kg), significantly decreased the immobility time of CUMS mice in the TST compared to the vehicle-treated control group (*t*-test, *p* < 0.001; Fig. 2C).

Two-way repeated measures ANOVA showed that none of the pharmacological manipulations or the CUMS procedure changed the animals’ locomotor activity [F(4,40) = 0.662, *p* > 0.05]. Furthermore, no drug x time interactions were observed [F(36,360) = 1.396, *p* > 0.05; Fig. 2D).

### Influence of a single and four-day treatment with M-5MPEP on mTOR, eEF2, TrkB, and SERT expression in the hippocampus in the CUMS model of depression

**Fig. 3:**
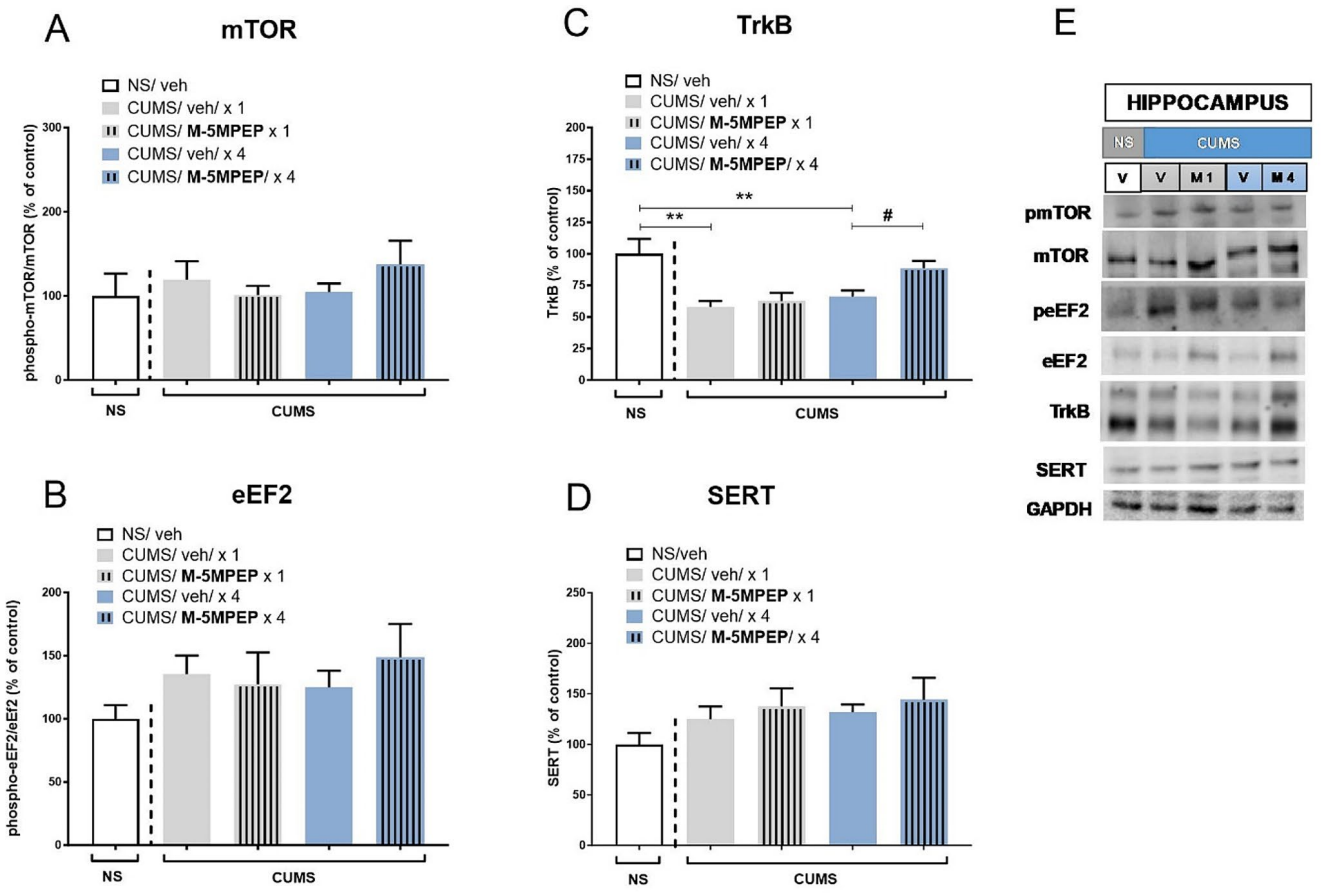
The effects of single and four-day administration of M-5MPEP (30 mg/kg) on the mTOR (**A**), eEF2 (**B**), TrkB (**C**), and SERT (**D**) levels determined by Western blot analysis in the synaptosome-enriched fraction of the hippocampus in the CUMS model of depression. (**E**) Exemplary immunoblots. The data were analyzed using one-way ANOVA followed by Tukey’s multiple comparisons test. Values (the mean ± SEM) are expressed as percentage of changes vs. control levels. ** *p* < 0.01 vs. the NS group; # *p* < 0.05 vs. a respective CUMS/vehicle group (*n* = 6–8). NS– non-stressed; CUMS– chronic unpredictable mild stress; V– vehicle; M1– single M-5MPEP administration; M4– four-day M-5MPEP administration

Statistical analyses of Western blots in the hippocampus did not reveal any differences in the phospho-mTOR/mTOR ratio between the vehicle NS group and CUMS group (*t*-test, *p* > 0.05). Furthermore, one-way ANOVA showed that M-5MPEP did not influence the phospho-mTOR/mTOR ratio [F(3,32) = 0.756, *p* > 0.05] (Fig. 3A). Hippocampal phospho-eEF2/eEF2 ratio was also unchanged in CUMS mice compared to NS controls (*t*-test, *p* > 0.05). However, a trend to increase the phospho-eEF2/eEF2 ratio in stressed animals was observed (Fig. 3B). One-way ANOVA did not reveal significant changes in the phospho-eEF2/eEF2 ratio between M-5MPEP-treated and vehicle-treated CUMS mice [F(3,34) = 0.261, *p* > 0.05] (Fig. 3B).

On the other hand, hippocampal TrkB level was significantly decreased in CUMS mice compared to NS animals (*t*-test, *p* < 0.01). Moreover, one-way ANOVA revealed that the TrkB level in the hippocampus was affected by M-5MPEP [F(3,34) = 6.47, *p* < 0.01]. Tukey’s post hoc test showed that four-day M-5MPEP treatment was required to reverse CUMS-induced changes in the TrkB level in the hippocampus (*p* < 0.05; Fig. 3C).

Finally, statistical analyses of SERT levels in the hippocampus showed no CUMS effect (*t*-test, *p* > 0.05) and lack of M-5MPEP treatment effect on the level of SERT in this structure in stressed animals [F(3,35) = 0.27, *p* > 0.01] (Fig. 3D).

### Influence of a single and four-day treatment with M-5MPEP on mTOR, eEF2, TrkB, and SERT expression in the PFC in the CUMS model of depression

**Fig. 4:**
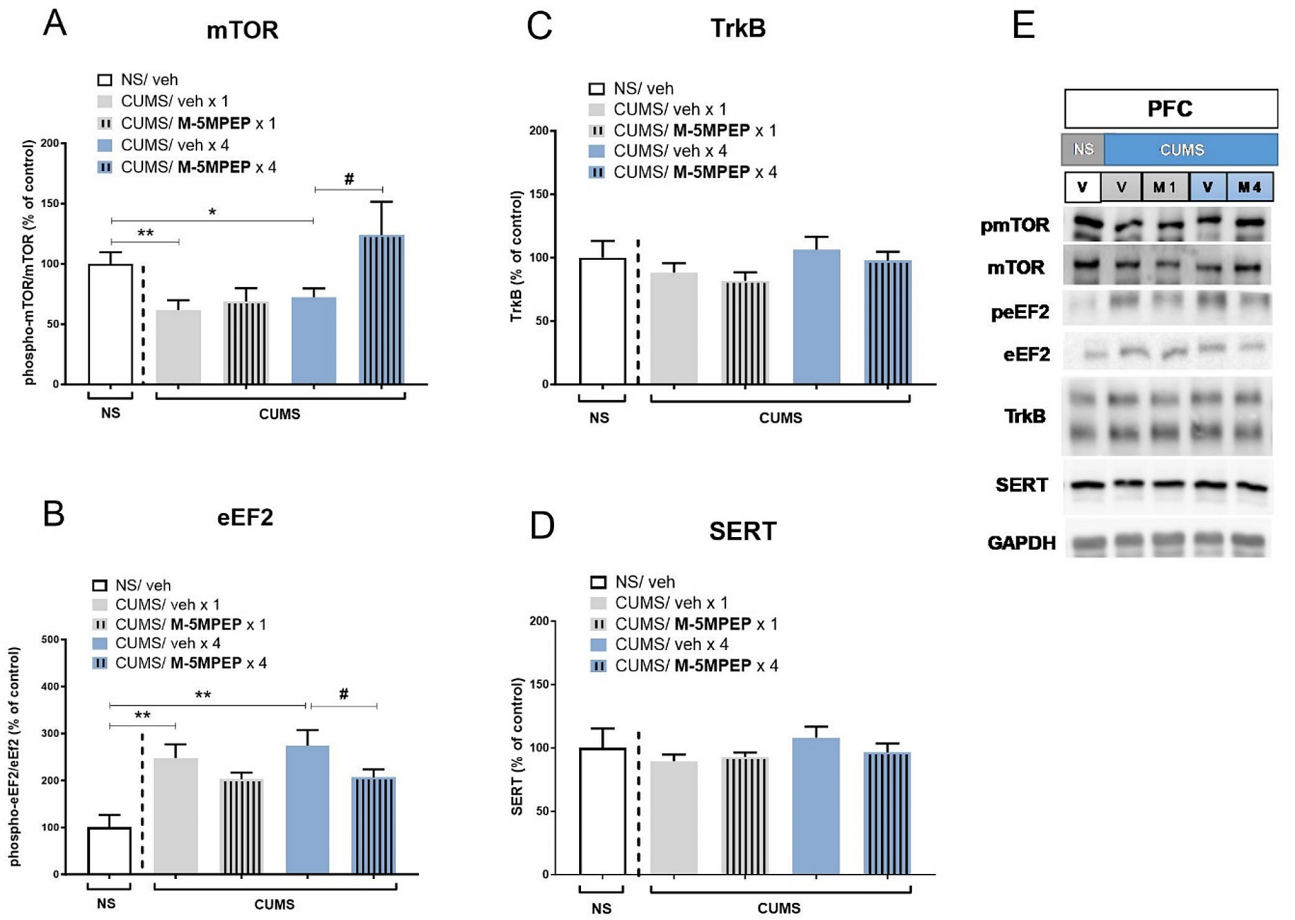
The effects of single and four-day administration of M-5MPEP (30 mg/kg) on the mTOR (**A**), eEF2 (**B**) TrkB (**C**), and SERT (**D**) levels determined by Western blot analysis in the synaptosome-enriched fraction of the PFC in the CUMS model of depression. (**E**) Exemplary immunoblots. The data were analyzed using one-way ANOVA followed by Tukey’s multiple comparisons test. Values (the mean ± SEM) are expressed as percentage of changes vs. control levels. * *p* < 0.01; ** *p* < 0.01 vs. the NS group; # *p* < 0.05 vs. a respective CUMS/vehicle group (*n* = 6–8). NS– non-stressed; CUMS– chronic unpredictable mild stress; V– vehicle; M1– single M-5MPEP administration; M4– four-day M-5MPEP administration

Western blot analysis showed that CUMS significantly reduced the phospho-mTOR/mTOR ratio compared to NS controls both in the single and four-day vehicle-treated group by 38% (*t*-test, *p* < 0.01) and 28%, (*t*-test, *p* < 0.05), respectively (Fig. 4A). One-way ANOVA revealed that M-5MPEP (30 mg/kg) induced a reversal of CUMS effect [F(3,31) = 4.751, *p* < 0.01] and Tukey’s post hoc test showed that four-day M-5MPEP treatment was required to produce a statistically significant effect (*p* < 0.05; Fig. 4A). Changes in the phosphor-eEF/eEF2 ratio in the CUMS mice compared to NS group were also found in the PFC. Phospho-eEF/eEF2 ratio was increased in vehicle-treated CUMS animals, both in single and four-day vehicle-treated groups, by 247% (*t*-test, *p* < 0.01) and 285% (*t*-test, *p* < 0.01), respectively (Fig. 4B). One-way ANOVA showed that M-5MPEP decreased CUMS-induced effects [F(3,32) = 4.013, *p* < 0.05]. Tukey’s post hoc test revealed statistical significance only for the four-day M-5MPEP treatment group (*p* < 0.05; Fig. 4B).

Statistical analyses of Western blots in the PFC did not reveal any significant changes in the level of TrkB between the vehicle-treated NS and CUMS groups (*t*-test, *p* > 0.05). One-way ANOVA revealed that the expression level of TrkB in CUMS animals was affected by M-5MPEP [F(3,36) = 3.102, *p* < 0.05]. However, Tukey’s post hoc test revealed no difference between M-5MPEP -treated vs. vehicle-treated CUMS mice, both after single and fourfold administration (*p* > 0.05; Fig. 4C). The expression level of SERT in the PFC was also unaffected by CUMS (*t*-test, *p* > 0.05). M-5MPEP did not change the SERT level in this brain structure in CUMS mice, both after single and fourfold administration [F(3,35) = 1,612, *p* > 0.05] (Fig. 4D).

### Evaluation of BDNF concentration in the PFC and hippocampus after single and four-day administration of M-5MPEP

**Fig. 5:**
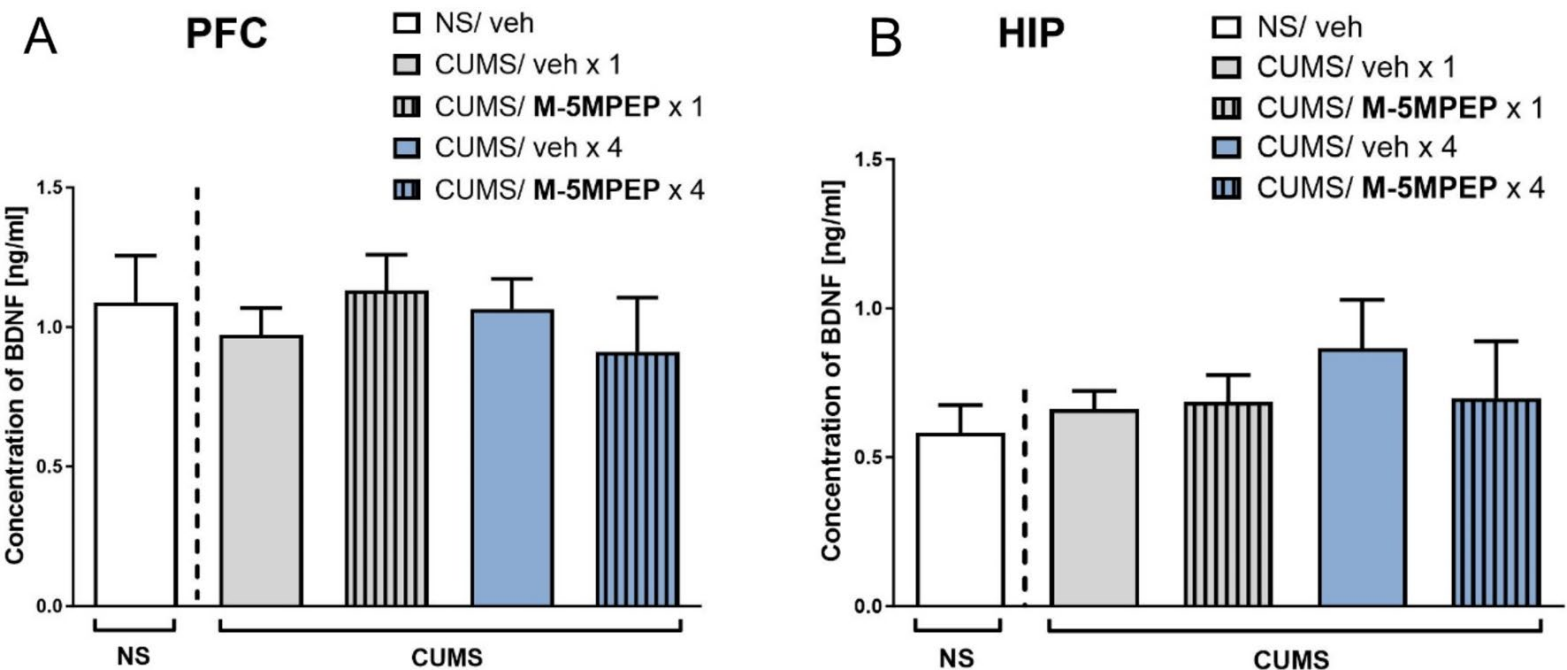
The effects of single and four-day administration of M-5MPEP (30 mg/kg) on BDNF levels in the PFC (**A**) and hippocampus (**B**). The values are expressed as the means ± SEM and were analyzed by one-way ANOVA followed by Tukey’s multiple comparisons test (*n* = 6–8). NS– non-stressed; CUMS– chronic unpredictable mild stress

Statistical analyses showed a lack of CUMS effect on the BDNF concentration in the PFC, compared to NS mice (*t*-test, *p* > 0.05). One-way ANOVA revealed that M-5MPEP did not affect the level of BDNF in the PFC, both after single and four-day administration in CUMS mice [F(3,27) = 0.508, *p* > 0.05] (Fig. 5A). In the hippocampus, no changes between vehicle-treated CUMS mice and vehicle-treated NS mice were found (*t*-test, *p* > 0.05). Furthermore, M-5MPEP did not influence hippocampal BDNF concentration in CUMS mice [F(3,24) = 0.444, *p* > 0.05] (Fig. 5B).

### Effects of M-5MPEP in the CUMS model of depression on field potentials (FPs) recording

**Fig. 6:**
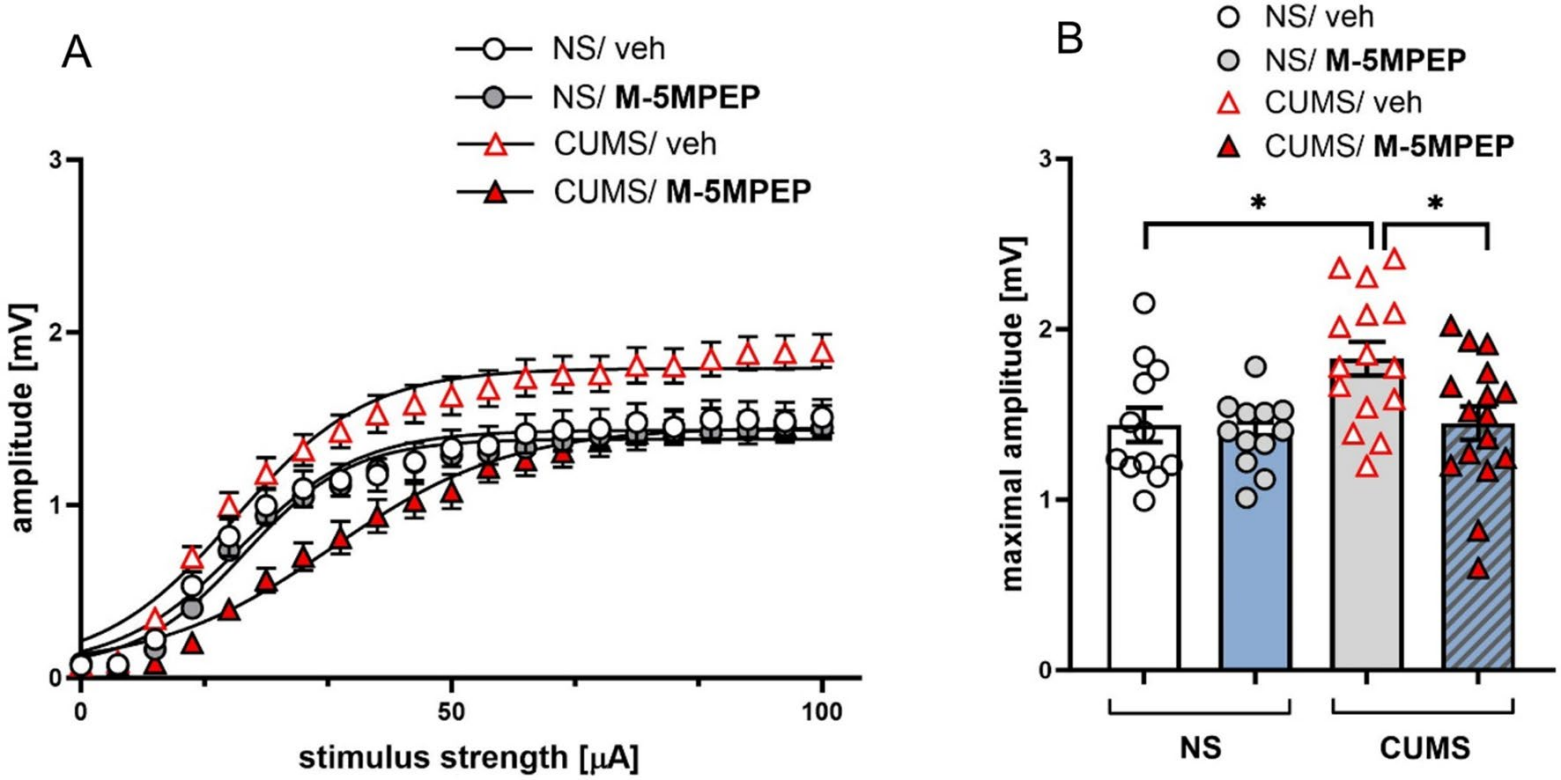
The effect of four-day administration of M-5MPEP (30 mg/kg) in the CUMS model of depression on field potential (FP) recording. (**A**) Effect of M-5MPEP in NS and CUMS mice on the relationship between stimulus intensity and the amplitude of FPs (mean ± SEM) recorded in mPFC slices originating from NS/veh animals (white circles), NS/M-5MPEP animals (gray circles), CUMS/veh animals (white triangles), and CUMS/M-5MPEP animals (red triangles); (**B**) Maximal FPs amplitudes in NS/veh, NS/M-5MPEP, CUMS/veh and CUMS/M-5MPEP groups. The values are expressed as the means ± SEM and were analyzed by two-way ANOVA followed by post hoc Tukey’s multiple comparisons test. * *p* < 0.05 vs. a respective control group. NS– non-stressed; CUMS– chronic unpredictable mild stress

Two-way ANOVA revealed significant effects of M-5MPEP and CUMS but no interaction on Boltzmann– calculated field potential amplitude [F(1,51) = 5.061, *p* < 0.05; F(1,51) = 5.582, *p* < 0.05 and F(1,51) = 3.039, *p* > 0.05, respectively). Tukey’s multiple comparisons test revealed a significant increase in the maximum evoked FP in slices obtained from animals subjected to CUMS, compared to those obtained from the non-stressed group (*p* < 0.05; Fig. 6B). Administration of M-5MPEP did not affect amplitude in the NS mice (*p* > 0.05) but it prevented the CUMS-induced increase in FP amplitude (Tukey’s multiple comparisons test; *p* < 0.05) and the results were comparable to slices obtained from non-stressed vehicle-treated group (*p* > 0.05) (Fig. 6B).

### Effects of M-5MPTP in the CUMS model of depression on LTP induction

**Fig. 7:**
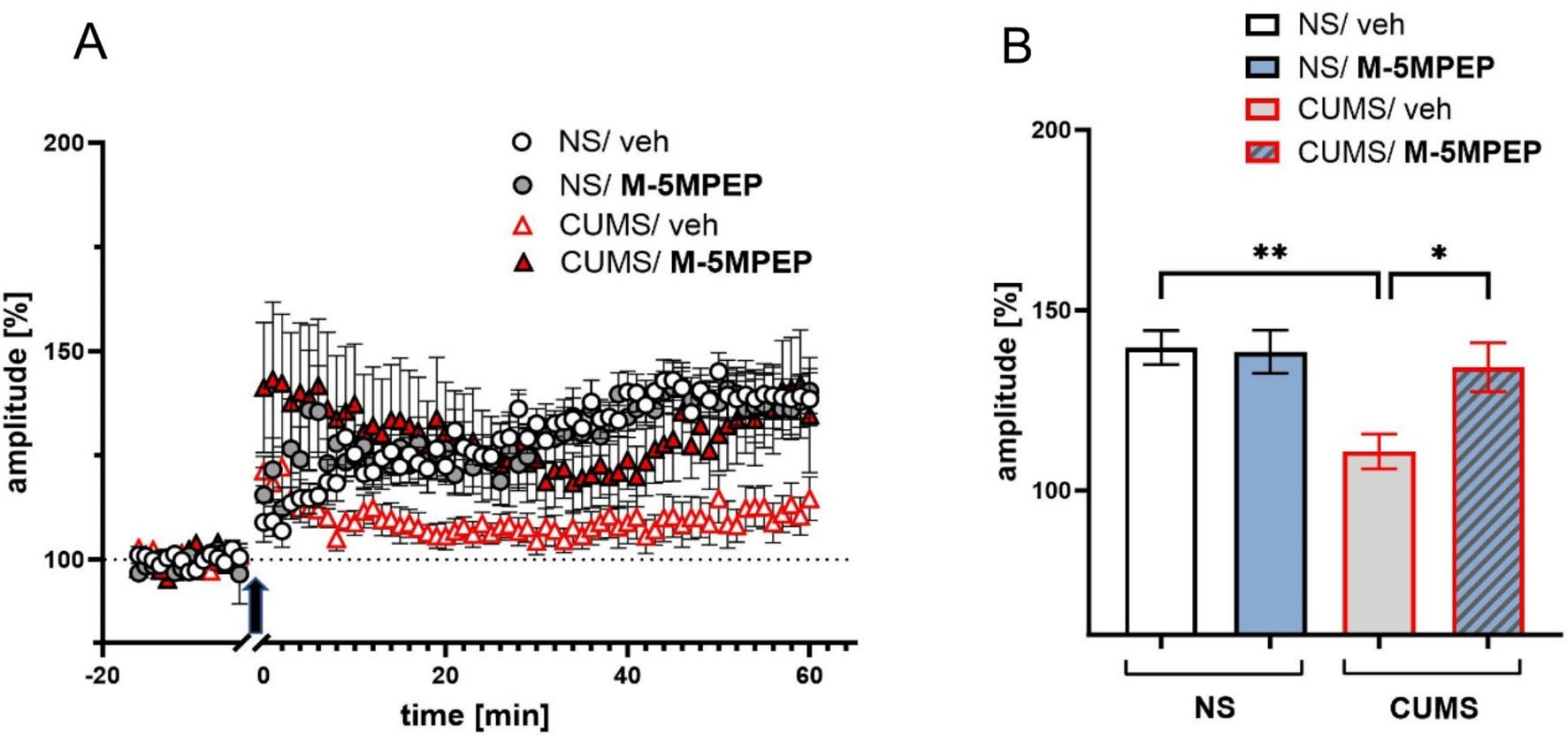
The effects of four-day administration of M-5MPEP (30 mg/kg) in the CUMS model of depression on LTP recorded in mPFC slices. (**A**) The plot of the amplitude of FPs (mean ± SEM) recorded in the recorded in mPFC slices originating from NS/veh animals (white circles), NS/M-5MPEP animals (gray circles), CUMS/veh animals (white triangles), and CUMS/M-5MPEP animals (red triangles); the arrow denotes the time of the beginning of TBS; (**B**) LTP magnitude in NS/veh, NS/M-5MPEP, CUMS/veh and CUMS/M-5MPEP groups. The values are expressed as the means ± SEM and were analyzed by two-way ANOVA followed by post hoc Tukey’s multiple comparisons test. * *p* < 0.05, ** *p* < 0.01 vs. a respective control group. NS– non-stressed; CUMS– chronic unpredictable mild stress

Two-way ANOVA revealed a significant M-5MPEP effect on LTP ([F(1,50) = 7.898, *p* < 0.01] and the CUMS x drug interaction [F(1,50) = 4.327, *p* < 0.05]. In slices prepared from non-stressed vehicle-treated animals, the mean amplitude of FPs measured between 60 and 65 min after TBS stimulation was 139 ± 1.2% of baseline. Tukey’s multiple comparisons test showed that LTP was significantly attenuated in slices prepared from CUMS animals (110 ± 1.2%, *p* < 0.01, Fig. 7B). There was no effect of M-5MPEP administration on LTP compared to control non-stressed animals (138 ± 1.4%, *p* > 0.05). However, administration of M-5MPEP reversed CUMS-induced effect on LTP (*p* < 0.05, Tukey’s test), and the magnitude of LTP was not different from that recorded in slices originating from non-stressed vehicle mice (134 ± 2.5%, *p* > 0.05, Fig. 7B).

## Discussion

In the present study, we demonstrate that partial NAM of the mGlu_5_ receptor, M-5MPEP, abolishes CUMS-induced behavioral effects in mice after short-term treatment, strongly confirming its rapid antidepressant, ketamine-like potential. This effect may be related to mTOR and eEF2 activity changes and modulation of glutamate transmission and LTP in the mPFC.

CUMS is a well-validated and widely used model of depression (Willner 2017), allowing for the determination of the action of RAADs. In contrast to classical monoamine-based ADs, which show their antidepressant-like effect in this model after several weeks of treatment, thus reflecting the profile of action in humans, RAADs act quickly in the CUMS model after a single or short-term administration (Papp et al. 2017; Sun et al. 2016). Our recent study showed that short-term, four-day (but not single) administration of M-5MPEP (30 mg/kg) can produce prolonged antidepressant-like effects in mice 24 h after the last administration (Pałucha-Poniewiera et al. 2024). In the present study, we decided to investigate whether M-5MPEP, used in the same dose and schedule, would influence the behavioral changes induced by chronic stress. It was found that four-day, but not single, administrations of M-5MPEP (30 mg/kg) effectively abolished apathy-like symptoms of reduced grooming in the splash test, comparable to subanesthetic *(S)*-ketamine. Similar results were obtained in the SPT, in which stressed mice showed anhedonia, manifested by a decrease in 1% sucrose solution consumption, and four administrations of M-5MPEP influenced the reversal of this effect. Moreover, a significant M-5MPEP-induced decrease in immobility time in CUMS mice was observed in the TST after single and four administrations. Finally, no significant changes were observed in the locomotor activity test between the stressed and non-stressed groups and between the groups receiving the compound and the vehicle-treated control groups. Importantly, using the same model of depression in the same strain of mice, we have previously shown in our lab that three days of administration of the classic SSRI antidepressant, fluoxetine, did not affect stress-induced behavioral changes. In contrast, ketamine, used in the same experiment, reversed CUMS-induced effects after a 3-day injection (Pałucha-Poniewiera et al. 2021). On the other hand, chronic, six-week treatment with fluoxetine induced a reversal of the stress-induced behaviors in this depression model, showing the therapeutic efficacy of fluoxetine after chronic treatment (Szewczyk et al. 2019). Therefore, reversing the effect of stress-induced changes in the CUMS model after 4 days of M-5MPEP administration presented in this study may indicate the rapid therapeutic M-5MPEP action, resembling the RAADs effect.

Explanation of the mechanism responsible for the rapid and prolonged antidepressant effect is one of the most actively studied areas of psychopharmacology. These studies mainly involve ketamine, the only approved RAAD so far (Mahase 2019). As stated in the introduction, among the mechanisms considered, two main theories come to the fore: the disinhibition theory assuming a crucial role of mTOR cascade activation (Li et al. 2010), and the homeostatic synaptic plasticity hypothesis, which draws attention to the role of the eEF2 factor and BDNF/TrkB signaling (Autry et al. 2011; Kim et al. 2023). Based on the above hypotheses, we investigated if M-5MPEP influenced mTOR and eEF2 protein levels in the hippocampus and PFC in CUMS animals. For both proteins, the ratio of phosphorylated to unphosphorylated form was analyzed.

Western blot analyses of the hippocampal tissue did not reveal any differences in the levels of phosphorylated forms of mTOR or eEF2 between the stressed and non-stressed groups. Furthermore, no effect of M-5MPEP on the levels of these factors was observed in CUMS animals. However, a significant decrease in the level of TrkB protein, which is a receptor for BDNF, was observed in CUMS mice compared to non-stressed mice. Moreover, a reversal of this effect after four administrations of M-5MPEP was also observed, suggesting this factor’s involvement in the tested compound’s behavioral effects. Nevertheless, using the Elisa method, the measurement of BDNF levels in the hippocampus did not reveal any changes between the studied groups. However, the BDNF measurement method might not be appropriate for detecting its changes, especially since the tissue was collected three days after the last administration when the action of M-5MPEP could not directly influence the level of BDNF itself. On the other hand, changes in the level of its receptor, which are adaptive, may indicate the role of BDNF in this process. Importantly, our previous study showed that the prolonged antidepressant effect of M-5MPEP in non-stressed mice (24 h after the last of four injections) was blocked by a TrkB receptor antagonist, ANA-12, which also indicates the role of the TrkB-BDNF signaling in the mechanism of the sustained action of M-5MPEP (Pałucha-Poniewiera et al. 2024). An experiment consisting in blocking the behavioral effect of M-5MPEP by a TrkB antagonist, which was not done because of a lack of approval of the ethics committee due to failure to meet the requirement of the 3R principles, would undoubtedly help to finally verify the hypothesis about the dependence of the antidepressant-like effect of M-5MPEP on the activation of the BDNF-TrkB signaling in the CUMS model of depression.

Interestingly, Western blot analyses of the PFC tissue showed opposite results. No changes in TrkB levels were observed between the NS and CUMS groups, and no effect of M-5MPEP on the level of this receptor protein in stressed mice was observed. However, significant changes were demonstrated in the levels of phosphorylated forms of both mTOR and eEF2. The level of mTOR in this structure was significantly lower in CUMS mice compared to non-stressed animals. However, in tissues obtained from stressed mice after four administrations of M-5MPEP, which had been effective in inducing antidepressant action, a reversal of this effect was observed, which means that the direction of mTOR changes was consistent with mechanisms characteristic of ketamine (Li et al. 2010).

In turn, the level of the phosphorylated form of eEF2 was significantly increased in the PFC of mice subjected to the CUMS procedure compared to the unstressed control. This result confirms the study of Babii et al. (2024), who showed a significant increase in phospho-eEF2 in the PFC (but not in the hippocampus) of mice in the CUMS model. Here, we show that four administrations of M-5MPEP caused a significant reversal of this effect, consistent with the widely documented direction of changes in the eEF2 activity after ketamine administration (Autry et al. 2011). Therefore, each of the studied factors, defined as key in the previously mentioned main hypotheses explaining the mechanism of RAAD action (namely mTOR and eEF2), may play a role in sustained M-5MPEP action.

Serotonergic transporter (SERT) protein level was also examined in both the hippocampus and PFC, given the earlier evidence for the involvement of the 5-HT system in the mechanism of action of RAADs (du Jardin et al. 2016; Gigliucci et al. 2013). In this study, we found no changes in SERT levels, either induced by CUMS or by the applied partial mGlu_5_ NAM. It is worth mentioning that our previous study showed that four-day M-5MPEP administration causing prolonged antidepressant-like effects in mice did not affect the SERT level in non-stressed mice. Furthermore, the antidepressant-like effects of M-5MPEP after acute administration in mice were not blocked by any serotonin (5HT) receptor antagonists, and M-5MPEP did not enhance the antidepressant-like effect of an SSRI, fluoxetine (Pałucha-Poniewiera et al. 2024). Therefore, it seems that the antidepressant-like action of M-5MPEP is unrelated to the activation of the 5-HT system, although this issue requires further research.

It has been found that medial PFC (mPFC) pyramidal neurons are impaired under chronic stress exposure (Radley et al. 2004). On the other hand, the antidepressant effect of RAADs correlates with the modulation of glutamatergic transmission and the enhancement of neuroplasticity processes in this structure (Li et al. 2010; Mingardi et al. 2023; Sala et al. 2022).

Therefore, we decided to use electrophysiological methods to examine the effect of four-day treatment with M-5MPEP on CUMS-induced changes in glutamatergic transmission in the mPFC and the effect on stress-induced impairments in LTP.

We found the increased amplitude of field potentials in the mPFC in slices obtained from CUMS mice compared to NS controls indicating stress-induced enhancement of glutamatergic transmission. This result confirms our previous observations in the CUMS model (Pałucha-Poniewiera et al. 2024) and is consistent with numerous data showing enhanced glutamatergic transmission in the PFC as a result of various long-stress procedures (Mingardi et al. 2023; Popoli et al. 2011; Sowa et al. 2015). Notably, a reversal of the stress-induced effect on excitatory synaptic transmission in the PFC by subanesthetic ketamine has been previously demonstrated. For example, ketamine antagonized stress-induced enhancement of glutamate release in the PFC in rats subjected to the footshock stress while inducing a mild increase of glutamate release in non-stressed individuals (Sala et al. 2022). In another study, ketamine treatment restored changes in glutamate release induced by CMS in rats, and the effect correlated to susceptibility to the stress procedure (Hu et al. 2023). Mingardi et al. (2023) also showed that CMS in rats increased the activity-dependent presynaptic glutamate release in the mPFC of vulnerable rats, and subanesthetic ketamine rescued this change. Here, we present an inhibitory effect of partial NAM mGlu_5_ on the stress-induced increased amplitude of field potentials in the mPFC, the action consistent with the previously demonstrated mechanism of subanesthetic ketamine. Interestingly, persistent elevations in extracellular glutamate concentrations in depression-related brain regions, including the PFC and hippocampus, are likely to be involved in the pathogenesis of depression (Musazzi et al. 2012), and repeated administration of amine-based antidepressants may also result in its reduction (Bobula et al. 2003, 2011; Gołembiowska and Dziubina 2000).

It is believed that enhancing glutamatergic transmission under chronic stress plays a critical role in the modulation of the neuroplasticity processes (McEwen et al. 2016).

Thus, in the next phase of electrophysiological studies, we investigated the effect of CUMS on the LTP, which correlates with the neuroplasticity induction. We showed that in cortical slices obtained from mice subjected to CUMS, the magnitude of LTP was significantly lower compared to slices obtained from unstressed mice, which suggests that this neuroplasticity-related process is impaired under the influence of chronic stress. This result confirms our previous observations using the CUMS model of depression (Pałucha-Poniewiera et al. 2023), as well as the data provided by other authors who indicated impaired LTP induced by different types of chronic stress, including CMS in rats and CSDS in mice (Burgdorf et al. 2015; Yang et al. 2018). Importantly, it was shown that single or short-term treatment with RAADs (e.g., rapastinel or *(R)*-ketamine coadministered with mGlu_2/3_ receptor antagonists) contributed to the reversal of this effect and the restoration of LTP magnitude in the PFC (Burgdorf et al. 2015; Pałucha-Poniewiera et al. 2023). A similar mechanism has been found in the hippocampus after ketamine, *(S)*-ketamine or (2R,6R)-HNK treatment (Aleksandrova et al. 2020; Yang et al. 2018). Here, we show that M-5MPEP administered for four consecutive days reversed the stress-induced decrease in LTP to control values but did not affect the LTP level in unstressed mice. These electrophysiological data indicate that partial mGlu_5_ NAM may mimic the effects induced by short-term treatments with RAADs. The mechanisms behind these effects might be explained by an “activity-dependent plasticity model” relating to the rapid antidepressant effect, which proposes that ketamine, which increases extracellular glutamate release after a single administration, induces disinhibition of GABAergic interneurons by a selective block of NMDA receptors and produces a subsequent increase in synchronous neuronal activity in the PFC, particularly in the gamma frequency band (30–80 Hz). These high-frequency oscillations activate endogenous activity-dependent synaptic strengthening mechanisms, including NMDA-receptor-dependent LTP (Thompson 2023). Verification of this hypothesis regarding the action of M-5MPEP would require further research.

Interestingly, our recent study showing the effect of *(R)*-ketamine coadministered with a mGlu_2/3_ antagonist on reversing the CUMS-induced decline in LTP in the PFC indicates that this effect correlates with the improvement of memory processes impaired by chronic stress (Pałucha-Poniewiera et al. 2023), which may be another argument for investigating M-5MPEP for its therapeutic application.

To sum up, our study indicates for the first time that the partial mGlu_5_ receptor NAM, M-5MPEP, abolishes the behavioral effects of long-term stress related to apathy and anhedonia, like subanesthetic *(S)*-ketamine after short-term administration. The mechanism of this action may depend on BDNF activity in the hippocampus, where we observed changes in the level of its receptor (TrkB). However, the primary brain region responsible for the action of M-5MPEP seems to be the PFC, where we showed CUMS-induced changes in the levels of two factors involved in the regulation of protein translation during neuroplasticity processes, namely mTOR and eEF2, and a reversal of these effects in mice administered with M-5MPEP in a therapeutically effective regimen. Using electrophysiological methods, we also showed evidence for the involvement of glutamatergic transmission in this mechanism, which is associated with regulating LTP, a process strongly correlated with enhancing neuronal plasticity processes in the PFC.

Based on the data presented here demonstrating the potential antidepressant activity of the partial mGlu_5_ receptor NAM in the CUMS model of depression and the previous results indicating that, unlike the full NAMs of this receptor, M-5MPEP does not exhibit noticeable adverse effects, we conclude that M-5MPEP deserves consideration in further studies as a new safe and effective RAAD. However, a limitation of this study resulting from the limited possibilities of carrying out the project is that it does not include females and uses only males in the experiments. At the same time, it is known that depression is almost twice as common in women as in men. Therefore, determining possible sex differences in the observed effects of M-5MPEP requires further studies using animals of both sexes.

## Electronic supplementary material

Below is the link to the electronic supplementary material.

Supplementary Material 1
