# Supplementary material for: Depression-like effects induced by chronic unpredictable mild stress in mice are rapidly reversed by a partial negative allosteric modulator of mGlu5 receptor, M-5MPEP
Source: Psychopharmacology (Berl). 2024 Nov 30;242(6):1259–73. doi: 10.1007/s00213-024-06724-4 (PMC12084170; doi:10.1007/s00213-024-06724-4)
Supplement: Supplementary file 1 — Supplementary Material 1 [file 213_2024_6724_MOESM1_ESM.docx]

**Supplementary data:**

**Synthesis of 2-((3-methoxyphenyl)ethynyl)-5-methylpyridine (M-5MPEP)**

The M-5MPEP was prepared using Shonogashira cross coupling reaction between commercially available 1-ethynyl-3-methoxybenzene (provided by CombiBlocks, QA-7338) and 2-iodo-5-methylpyridine (provided by CombiBlocks, QH-3318) , according to methods described by Alagille et al. (2005) and in PCT Int. Appl., 2005094822, 13 Oct 2005.

**Fig S1.** Reaction scheme.

**Procedure**

2-iodo-5-methylpyridine (0.80 g, 3.65 mmol, 1.0 eq,), 1-ethynyl-3-methoxybenzene (0.53 g, 4.01 mmol, 1.10 eq), PdCl2(PPh3)2 (precatalyst, 0,08 g, 0.11 mmol) and CuI (cocatalyst, 0.05 g, 0.26 mmol) were suspended in 3 mL of dry acetonitrile, degassed with Ar and treated with triethylamine (0.56 g, 5.53 mmol, 1.5 eq). The reaction was heated in a sealed tube at reflux for 16h. On completion, the reaction mixture was quenched with water and product was extracted with dichloromethane (3x), dried over MgSO4 and filtered. The solvents were removed in vacuo. The purification was carried out by column chromatography on silica gel using ethyl acetate and hexane as an eluate. There was obtained: 630 mg of solid 2-((3-methoxyphenyl)ethynyl)-5-methylpyridine (M-5MPEP), yield: 77%.

**Analytical data**

**LC-MS:** 100% (Rt = 3.01), ESI(+) m/z found: 224.83 [M+H]^+^. Molecular Weight calc’d for C_15_H_13_NO = 223.27.

**UPLC/MS**

UPLC/MS analysis was performed on Waters TQD spectrometer combined with UPLC Acquity H-Class with PDA eLambda detector. Waters Acquity UPLC BEH C18 1.7 μm 2.1 x 50 mm chromatographic column was used, at 40 °C, 0.3 mL/min flow rate and 1.0 μL injection volume (the samples were dissolved in LC-MS grade acetonitrile, typically at a concentration of 0.1–1 mg/mL prior to injection). All mass spectra were recorded under electrospray ionization in positive mode (ESI+) and chromatograms were recorded with UV detection in the range of 190–300 nm. The gradient conditions used were: 80% phase A (water + 0.1% formic acid) and 20% phase B (acetonitrile + 0.1% formic acid) to 100% phase B (acetonitrile + 0.1% formic acid) at 3.0 minutes, kept till 3.5 minutes, then to initial conditions until 4.0 minutes and kept for additional 2.0 minutes. Total time of analysis – 6.0 minutes.


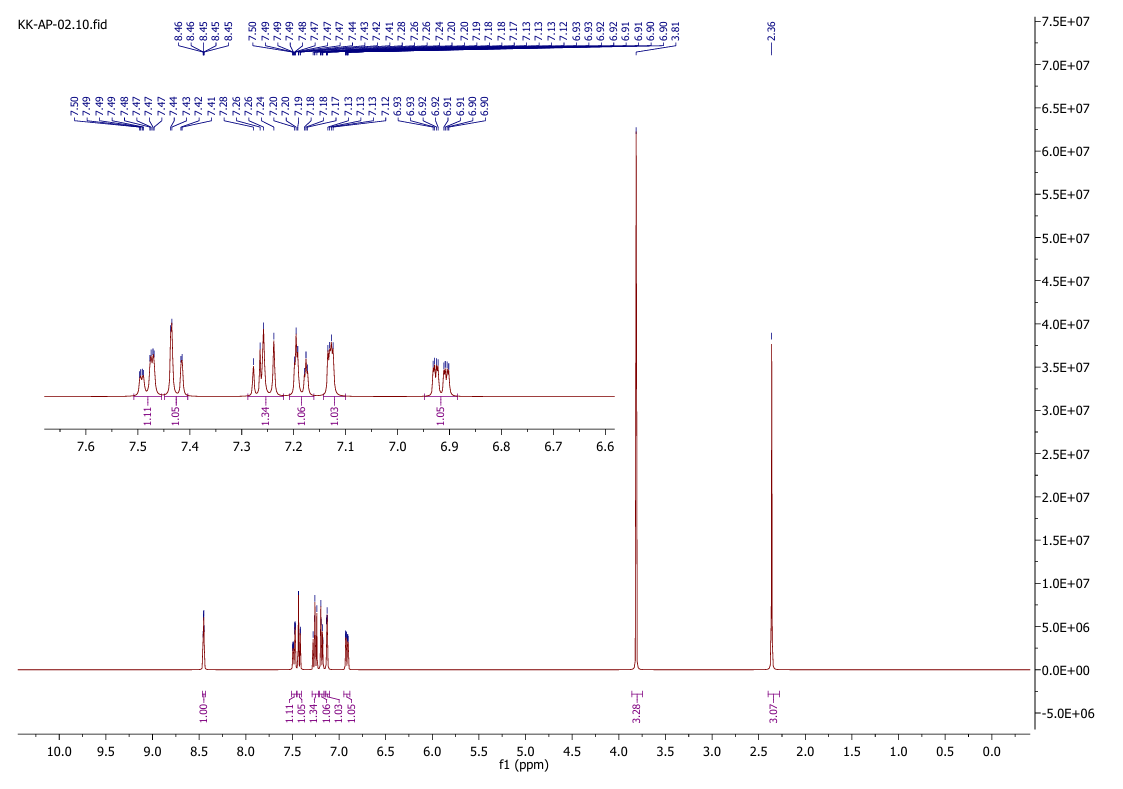


**Fig S2.** ^1^HNMR spectra of M-5MPEP synthesized in IF PAN, batch number: KK-AP-02.

**^1^H NMR** (400 MHz, Chloroform-d) δ ppm: 8.45 (dt, *J* = 2.0, 0.8 Hz, 1H), 7.48 (ddd, *J* = 8.0, 2.3, 0.8 Hz, 1H), 7.43 (dd, *J* = 8.0, 1.0 Hz, 1H), 7.26 (t, *J* = 7.7 Hz, 1H), 7.19 (dt, *J* = 7.6, 1.3 Hz, 1H), 7.13 (dd, *J* = 2.7, 1.4 Hz, 1H), 6.92 (ddd, *J* = 8.2, 2.7, 1.2 Hz, 1H), 3.81 (s, 3H), 2.36 (s, 3H).


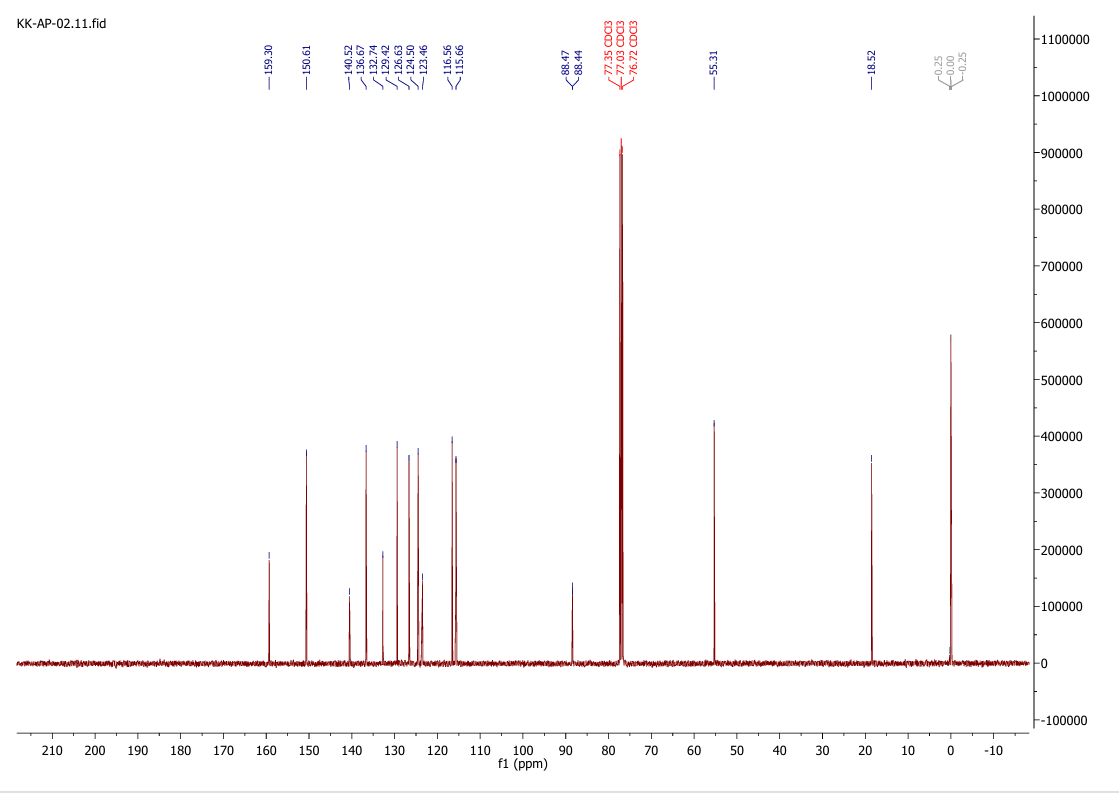


**Fig S3.** ^13^C NMR of M-5MPEP synthesized in IF PAN, batch number: KK-AP-02.

**^13^C NMR** (101 MHz, Chloroform-*d*) δ ppm: 159.30, 150.61, 140.52, 136.67, 132.74, 129.42, 126.63, 124.50, 123.46, 116.56, 115.66, 88.47, 88.44, 55.31, 18.52.

**NMR**

^1^H NMR spectra were measured at 400 MHz and ^13^C NMR spectra at 101 MHz on a Bruker (Ascend Evo 400 MHz) spectrometer in CDCl_3_ with TMS as an internal standard. The spectral data of M-5MPEP refer to its free bases. Chemical shifts are expressed in (ppm). Splitting patterns describe apparent multiplicities and are designated as s (singlet), d (doublet), t (triplet), q (quartet), and m (multiplet). Coupling constants are given in units of hertz (Hz). The 1HNMR and 13CNMR data were compared to literature data and confirmed the structure.

**References:**

Alagille D, Baldwin RM, Roth BL, Wroblewski JT, Grajkowska E, Tamagnan GD (2005) Synthesis and receptor assay of aromatic–ethynyl–aromatic derivatives with potent mGluR5 antagonist activity. Bioorg Med Chem 13:197-209.
